# Swadamshtradi rasayana attenuates radiofrequency radiation-induced oxidative stress and improves sperm parameters in Swiss albino mice

**DOI:** 10.3389/frph.2026.1912688

**Published:** 2026-08-26

**Authors:** Vahab Rahimkutty Abdul, Meera Sudha, Neenu Prakash Easwaramangalath, Delvin T. Robin, Anusree Dileep, Vandana Rani Madhavan

**Affiliations:** Department of Swasthavritta (Social and Preventive Medicine), Amrita School of Ayurveda, Amritapuri, Amrita Vishwa Vidyapeetham, Kollam, India

**Keywords:** ayurveda, male infertility, oxidative stress, radiofrequency electromagnetic radiation, sperm DNA damage, swadamshtradi rasayana

## Abstract

**Introduction:**

Exposure to radiofrequency electromagnetic radiation (RF-EMR) has been linked in several studies to male reproductive dysfunction, with oxidative stress and sperm DNA damage proposed as key underlying mechanisms.

**Methods:**

The present study evaluated the protective effect of Swadamshtradi rasayana (SDR) against RF-EMR–induced sperm alterations in mice. Twenty-four Swiss albino mice were allocated into four groups: control, RF-EMR–exposed, RF-EMR with Swadamshtradi rasayana (729 mg/kg, orally), and RF-EMR with *α*-tocopherol (32.4 mg/kg, orally). Animals were exposed to RF-EMR (900–1800MHz) for 3 h/day for 35 days, and sperm count, viability, reactive oxygen species levels, antioxidant enzyme activity, lipid peroxidation, and DNA fragmentation were assessed.

**Results:**

RF-EMR exposure was associated with a significant reduction in sperm count and viability and a significant increase in ROS levels, lipid peroxidation, and DNA fragmentation (*p* < 0.0001 compared with control). Administration of Swadamshtradi rasayana significantly improved sperm parameters and attenuated oxidative stress compared with the RF-EMR–exposed group (*p* < 0.0001).

**Conclusion:**

These findings suggest that Swadamshtradi rasayana may mitigate RF-EMR-induced oxidative stress and associated sperm damage. The observed protective effects are consistent with the reported antioxidant properties of its constituent herbs.

## 1 Introduction

Infertility affects about 15% of couples of reproductive age worldwide and has emerged as a major public health concern. An estimated 60–80 million couples experience infertility each year, including an estimated 15–20 million couples in India (1). Male factors contribute to about 50% of infertility cases (2). Male infertility is commonly associated with abnormalities in semen parameters such as reduced sperm count, impaired sperm motility, and abnormal sperm morphology (3). Recent global studies have revealed a progressive decline in male reproductive health, including a 51.6% reduction in sperm concentration between 1973 and 2018 (4). Environmental pollutants, endocrine-disrupting chemicals, and lifestyle changes have been implicated in this decline (5). The rapid expansion of wireless communication technologies has increased human exposure to radiofrequency electromagnetic radiation (RF-EMR). Mobile communication systems operating typically within the 900–1800MHz range contribute to continuous RF-EMR exposure in daily life (6). Such exposure has been associated with adverse biological effects. The testes are considered particularly vulnerable because of their relatively limited protective barriers. RF-EMR has been reported to adversely affect the male reproductive system and impair sperm quality (7, 8).

Male infertility is a multifactorial syndrome involving metabolic abnormalities, post-testicular defects, immunological disturbances, lifestyle factors, and environmental exposures (9). It arises from the disruption of multiple physiological processes, including oxidative stress and sperm dysfunction (10). Oxidative stress plays a central role in RF-EMR–induced reproductive damage by promoting the excessive generation of reactive oxygen species, leading to lipid peroxidation, mitochondrial dysfunction, and sperm DNA damage (10). A recent study reported that RF-EMR-induced testicular injury may involve inflammatory and histopathological alterations, including changes in cytokine expression, indicating that mechanisms beyond oxidative stress, such as alterations in the testicular immune microenvironment, may also play a role (11).

A considerable proportion of male infertility cases, estimated between 37%–58%, are classified as idiopathic or unexplained infertility based on routine semen analysis (12, 13). Semen analysis may reveal abnormalities such as reduced sperm count, motility, and morphology (14). In addition, sperm DNA damage, elevated reactive oxygen species (ROS), antisperm antibodies, and sperm dysfunction have been identified as major contributing factors to male infertility (3, 12, 15).

Spermatogenesis is highly dependent on the timely action of intratesticular testosterone (16). However, administration of exogenous testosterone and androgenic medications may suppress the hypothalamic–pituitary–gonadal axis and impair spermatogenesis (17). Certain medications, including antihypertensive and psychotropic drugs, may also adversely affect sperm production and motility (18). These limitations emphasize the need for safer and more effective therapeutic approaches for male infertility.

Herbal medicines have long been used as alternatives to synthetic drugs due to their relatively lower toxicity and traditional use in infertility management (19, 20). Despite growing evidence implicating oxidative stress in RF-EMR–induced sperm damage, most studies have focused on individual antioxidants or isolated phytoconstituents. Evidence on the effects of polyherbal formulations, particularly classical Ayurvedic rasayana preparations, on sperm DNA integrity and oxidative stress–mediated reproductive dysfunction remains limited. In this context, classical Ayurvedic formulations containing herbs with documented antioxidant potential may offer a multitarget therapeutic approach.

In Ayurveda, the Astangahridaya (Uttarasthana, Rasayana Vidhi Adhyaya) mentions Swadamshtradi yoga, which consists of powdered *Tribulus terrestris* (Gokshura), *Phyllanthus emblica* (Amalaki), and *Tinospora cordifolia* (Guduchi) mixed with ghee and honey. Regular consumption of this formulation is indicated for enhancing vitality, promoting reproductive health, and improving longevity (21). These constituent herbs are reported to possess antioxidant and free radical scavenging properties, which may be relevant to mitigating oxidative stress–mediated sperm damage (22–24). Experimental studies have also demonstrated the radioprotective effects of *Phyllanthus emblica* and *Tinospora cordifolia* against radiation-induced cellular damage (25, 26), suggesting their potential role in preserving cellular integrity under oxidative stress conditions. Additionally, *Tribulus terrestris* extract has been reported to improve sperm count and progressive motility (24), indicating a possible role in supporting sperm function. Therefore, the present study investigated the effect of Swadamshtradi rasayana (SDR) on RF-EMR–induced reproductive alterations by assessing oxidative stress, sperm DNA damage, and sperm functional parameters in Swiss albino mice.

## 2 Materials and methods

### 2.1 Animal care

Twenty-four male Swiss albino mice (25–30 g) were used in the study. All experimental procedures were approved by the Institutional Animal Ethics Committee (IAEC) of Rajiv Gandhi Centre for Biotechnology (RGCB), Thiruvananthapuram, India (RGCB Animal Facility; 326/GO/ReBiBt/S/2001/CPCSEA, IAEC/734/PRK/2019). Animals were maintained at a controlled room temperature of 22 ± 2 °C and relative humidity 50 ± 10% under a 12:12 h light/dark cycle. Animals were provided with free access to standard chow and water. All procedures were carried out in accordance with the National Institutes of Health guidelines for the care and use of laboratory animals (27).

### 2.2 Preparation of Swadamshtradi rasayana

The raw herbal materials used in the preparation of Swadamshtradi rasayana, namely Amalaki (*Phyllanthus emblica*), Guduchi (*Tinospora cordifolia*), and Gokshura (*Tribulus terrestris*), were procured from a licensed herbal drug supplier, Thiruvananthapuram, Kerala, India. The botanical identity of the plant materials was authenticated by Senior Scientist and Head, Plant Genetic Resources Division, KSCSTE–Jawaharlal Nehru Tropical Botanic Garden and Research Institute (JNTBGRI), Palode, Thiruvananthapuram, Kerala, India, and a certificate of botanical authentication was obtained prior to the study. Amalaki and Guduchi roots were cut into small pieces, thoroughly dried, powdered using a high-speed mechanical grinder, and sieved through ASTM mesh No. 80 to obtain a uniform fine powder. Gokshura was processed in the same manner. The quality of the ghee and honey used in the formulation was verified through physicochemical analysis at the Quality Control Laboratory, Arya Vaidya Sala, Kottakkal, Kerala, India, and Certificates of Analysis confirming compliance with the prescribed quality standards were obtained. The fine powders of Amalaki, Gokshura, and Guduchi were mixed in equal proportions (50 g each). The formulation was administered along with ghee and honey as described in Astangahridaya. According to the Ayurvedic Pharmacopoeia of India (API), the recommended dosage of these ingredients in powder form is 3–6 g. The quantities of honey and ghee were maintained at twice those of the primary herbal ingredients (28).

### 2.3 Experimental design

Twenty-four male Swiss albino mice were allocated to four groups (*n* = 6 per group), according to the predefined study design, with each group housed in separate polycarbonate cages. Formal randomization was not implemented for group allocation. Within each group, spermatozoa from three animals were used for sperm functional analyses (sperm count and viability), while spermatozoa from the remaining three animals were used for oxidative stress and molecular analyses, including reactive oxygen species (ROS), lipid peroxidation (LPO), superoxide dismutase (SOD), and sperm DNA fragmentation. This approach ensured adequate biological material for all planned analyses while minimizing the number of animals used. The sample size was determined using the resource equation approach recommended for experimental animal studies to ensure both statistical validity and ethical use of animals. The error degrees of freedom (E = N−G) were 20, which falls within the recommended range of 10–20 for exploratory animal studies (29). Furthermore, the sample size was selected in accordance with the principles of the 3Rs (Replacement, Reduction, and Refinement), ensuring the use of the minimum number of animals necessary to obtain scientifically valid and reproducible results (30). The control group received the standard diet without RF-EMR exposure or any experimental treatment. The EMR group was exposed to RF-EMR at a dual-band frequency of Enhanced Global System for Mobile Communications (EGSM) 900/1800MHz for 3 h/day. The EMR + SDR group received Swadamshtradi rasayana (729 mg/kg, orally) along with RF-EMR exposure. The EMR + AT group served as the positive control and received *α*-tocopherol (32.4 mg/kg, orally) along with RF-EMR exposure at the same frequency and duration. Animals in the control and RF-EMR groups received an equivalent volume of distilled water by oral gavage as the vehicle control, thereby ensuring that all experimental groups underwent comparable handling and procedural stress. The experimental period was 35 days. This duration was selected because it approximately corresponds to one complete spermatogenic cycle in mice, thereby enabling the evaluation of the cumulative effects of RF-EMR exposure and the protective effects of Swadamshtradi rasayana throughout the entire spermatogenic cycle (31, 32). The investigators performing sperm analysis, flow cytometry, and biochemical assays were not blinded to treatment allocation.

The mouse-equivalent doses of Swadamshtradi rasayana and *α*-tocopherol were determined from the therapeutic human dose using the body surface area conversion method (33). All treatments were administered orally once daily by oral gavage between 10:00 and 11:00 AM to minimize circadian variations. RF-EMR exposure was generated using a Nokia 105 mobile phone (107 × 44.8 × 14.3 mm; weight 70 g). The mobile phone was positioned centrally beneath each cage and maintained in continuous active call mode throughout the 3-hour exposure period to simulate sustained RF-EMR emission. Animals in the RF-EMR exposure groups were housed individually in standard polycarbonate cages and remained free-moving throughout the 3-hour exposure period; no restraint or immobilization was applied at any stage of the experiment. The mobile phone was positioned centrally beneath each cage at a fixed distance of approximately 3 cm from the cage base to maintain consistent proximity between the radiation source and the animals. Control animals were housed in a separate room within the same animal facility under comparable environmental conditions but without the presence of mobile phones or other RF-EMR sources. This served as the sham-control condition and prevented unintended RF-EMR exposure.

After 35 days of RF-EMR exposure, the animals were euthanized by cervical dislocation and the cauda epididymis was dissected. All dissection procedures were carried out at 37 °C. The cauda was carefully cleaned and minced in Hanks’ Balanced Salt Solution (HBSS) using a sterile lancet and incubated in a 5% CO₂ incubator at 37 °C for 5–10 min to allow spermatozoa to swim out from the epididymal tubules. The suspension was then filtered through an 80-µm Nitex membrane to separate sperm from tissue debris.

### 2.4 Sperm functional parameters

#### 2.4.1 Sperm count

Sperm samples were diluted with 3% saline to immobilize the spermatozoa, with a dilution factor of 1:20. The diluted sample was mixed thoroughly using a vortex for 10 s at maximum speed. A volume of 6.5 µL of the semen sample was dispensed into the haemocytometer and kept horizontally for 2–3 min at room temperature. The haemocytometer was examined using Nikon phase-contrast optics at ×200 magnification. A total of 200 spermatozoa were counted per replicate to obtain an acceptably low sampling error. Spermatozoa were counted in the four corner squares and the central square of the haemocytometer grid. The sperm concentration was estimated using a standard haemocytometer method as described in the WHO laboratory manual (34). The sperm count was calculated using the formula:

Meanspermcount×5×10,000×dilutionfactor

The sperm count was expressed as the number of spermatozoa per milliliter.

#### 2.4.2 Sperm viability

Sperm viability was assessed using the LIVE/DEAD® Sperm Viability Kit (Molecular Probes, Leiden, The Netherlands) according to the manufacturer's protocol. A concentration of 1–5 × 10⁶ spermatozoa suspended in HBSS was used. Samples were analyzed by flow cytometry using a FACSAria II (BD Biosciences). Excitation was performed at 488 nm. The emission of SYBR-14 fluorescence was detected using a 530/30 nm, 502 LP filter, and propidium iodide (PI) fluorescence was detected using a 585/42 nm, 556 LP filter. For each sample, 10⁴ events were recorded after gating to identify spermatozoa as live (SYBR-14 positive), dead (PI positive), or dying/moribund (SYBR-14 positive/PI positive) (35).

### 2.5 ROS assay

Reactive oxygen species (ROS) production in spermatozoa was assessed using 2′,7′-dichlorodihydrofluorescein diacetate (DCFDA) (Sigma-Aldrich, MO, USA). A concentration of 2–5 × 10⁶ spermatozoa suspended in HBSS was used for staining with DCFDA. The spermatozoa were incubated with DCFDA at a final concentration of 10 µg/mL in 50% ethanol in the dark for 15 min at 37 °C before analysis by flow cytometry using a FACSAria II (BD Biosciences). Excitation was performed at 488 nm, and fluorescence emission was detected using a 530/30 nm, 502 LP filter. A total of 10⁴ events were recorded for each sample, and the results were expressed as mean fluorescence intensity (MFI) (36).

### 2.6 DNA fragmentation analysis

#### 2.6.1 Sperm Chromatin Structure Assay (SCSA)

Sperm samples (50 µL) reconstituted in phosphate-buffered saline (PBS) were mixed with 100 µL of TNE buffer (0.001 M EDTA, 0.15 M NaCl, 0.01 M Tris-HCl; pH 7.4). The mixture was then treated with 400 µL of acid detergent solution (0.15 M NaCl, 0.12 N HCl, 0.1% Triton X-100; pH 1.2) and incubated at room temperature for 15 min. Subsequently, the samples were stained with 1.2 mL acridine orange (AO) staining solution (0.15 M NaCl, 0.126 M Na₂HPO₄, 0.037 M citric acid, 0.0011 M EDTA; pH 6.0; containing 6 mg/mL AO). After incubation for 3–5 min at room temperature, the sperm samples were analyzed by flow cytometry (BD Biosciences). The sperm chromatin structure assay was performed using a modified protocol (37, 38). The modifications included increasing the acid detergent concentration from 0.08 N to 0.12 N and reducing the reaction time from 30 min to 15 min to minimize self-fluorescence and improve discrimination between single- and double-stranded DNA.

A total of 5,000 events were recorded by flow cytometry. DNA denaturation was assessed using the DNA fragmentation index (DFI) based on the fluorescence emitted by acridine orange intercalated with single- and double-stranded DNA. Excitation was performed at 488 nm. The emission of green fluorescence, Fluorescein Isothiocyanate (FITC) was detected using a 530/30 nm, 502 LP filter, while red fluorescence [Phycoerythrin (PE)/Texas Red] was detected using a 616/23 nm, 610 LP filter.

Three populations were considered in the analysis: P2 (intact DNA), P3 (moderately fragmented DNA), and P4 (highly fragmented DNA). The DFI was calculated as:

DFI=(P3+P4)/(P2+P3+P4)

The definite DNA fragmentation index (DDFI) was calculated as:

DDFI=P4/(P2+P3+P4)

### 2.7 Biochemical analysis

#### 2.7.1 Superoxide dismutase (SOD) activity

Sperm pellets were reconstituted in chilled Tris–HCl buffer (50 mM, pH 8.2) with 1 mM EDTA. The spermatozoa were homogenized at 4 °C at 13,000 rpm for three cycles at 30 s intervals. The homogenates were treated with 1% Triton X-100 to obtain a final concentration of 0.2%, incubated at 4 °C for 30 min, and centrifuged at 14,000 rpm for 20 min at the same temperature. The resulting supernatant was used for the assessment of SOD activity based on the inhibition of pyrogallol auto-oxidation, with minor modifications (39). The auto-oxidation rate of pyrogallol was recorded at 420 nm. One unit of SOD activity was defined as the amount of enzyme required to achieve 50% inhibition of pyrogallol auto-oxidation. Percentage inhibition (%) was calculated as:

$$[(\Delta A_{420}/\min\_\mathrm{control}\;-\;\Delta A_{420}/\min\_\mathrm{sample})\;/\;\Delta A_{420}/\min\_\mathrm{control}]\;\times \;100$$

where *Δ*A₄₂₀/min_control represents the rate of increase in absorbance observed in the control reaction, whereas *Δ*A₄₂₀/min_sample denotes the rate in the presence of the sample.

#### 2.7.2 Malondialdehyde (MDA) levels

MDA levels were determined by the thiobarbituric acid reactive substances (TBARS) method described by Chari and Colagar (2011). Briefly, sperm samples suspended in HBSS buffer were homogenized at 4 °C for three cycles with 30 s intervals. After centrifugation, 100 µL of sperm supernatant was combined with 900 µL of distilled water. Thereafter, 0.5 mL of thiobarbituric acid reagent was introduced into the sperm suspension. The mixture was heated in a water bath for 1 h and subsequently cooled to room temperature. Following centrifugation at 4000 × g, the absorbance of the supernatant was recorded at 534 nm using a spectrophotometer. Lipid peroxidation levels were expressed as micromolar (µM) MDA based on an extinction coefficient of 155 mM⁻^1^ cm⁻^1^ (40).

### 2.8 Statistics

Statistical analyses were performed using GraphPad Prism version 8.0.2 (GraphPad Software, San Diego, CA, USA). Data normality was assessed using the Shapiro–Wilk normality test prior to statistical analysis. Datasets satisfying the assumption of normality, including sperm count, sperm viability, reactive oxygen species (ROS), malondialdehyde (MDA), and sperm DNA fragmentation indices (DFI and DDFI), were analyzed using one-way analysis of variance (ANOVA) followed by Tukey's multiple-comparisons test. For the superoxide dismutase (SOD) dataset, for which the assumption of normality was not satisfied in two experimental groups, the Kruskal–Wallis test followed by Dunn's multiple-comparisons test was used. Data are presented as individual observations with group mean ± SEM for normally distributed datasets and with median and interquartile range (IQR; 25th–75th percentile) for the non-parametric SOD dataset. A *p* value < 0.05 was considered statistically significant.

## 3 Results

### 3.1 Sperm functional parameters

**Figure 1:**
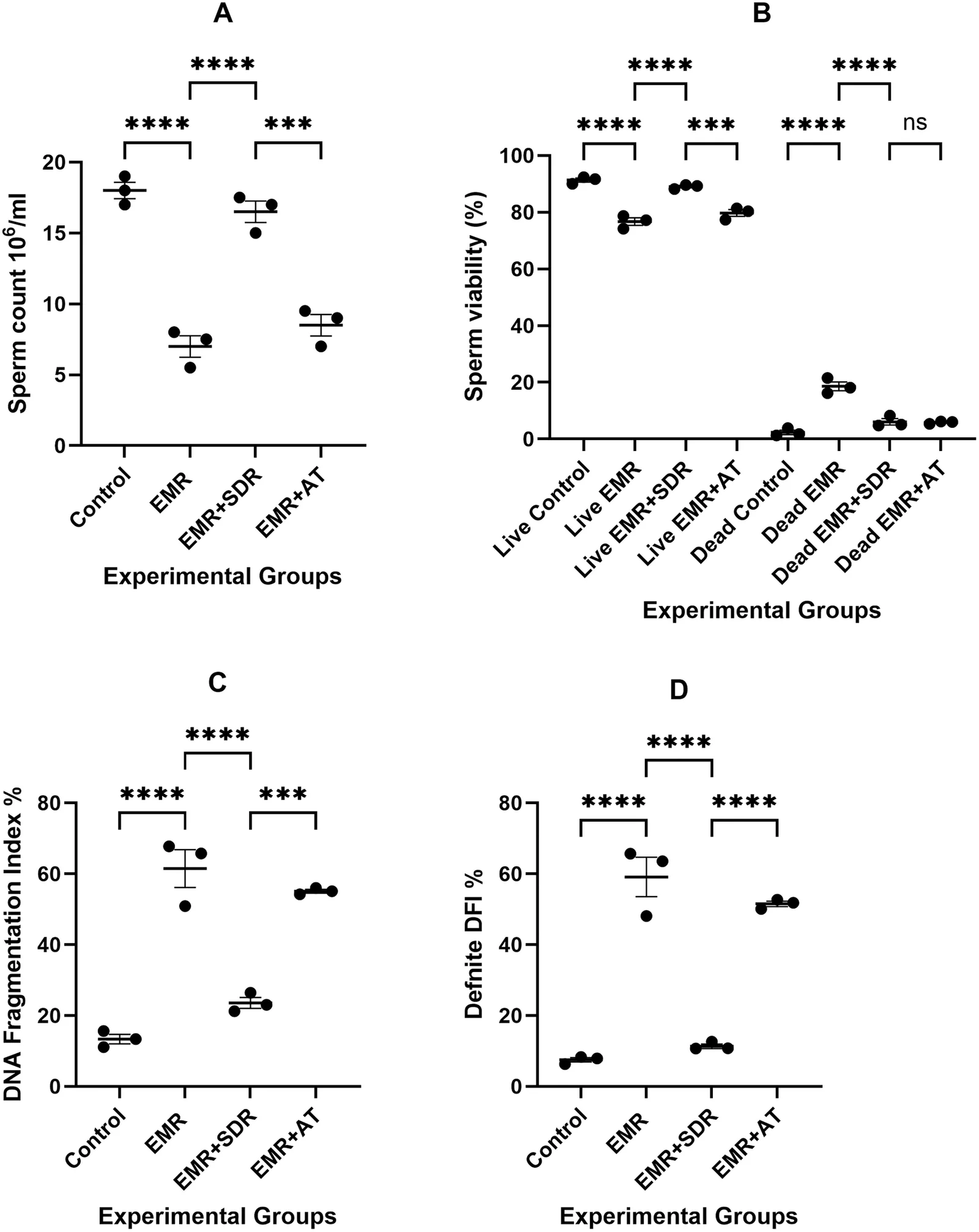
Effects of RF-EMR exposure and swadamshtradi rasayana treatment on sperm functional parameters in Swiss albino mice. **(A)** Sperm count, **(B)** sperm viability assessed by the LIVE/DEAD assay, **(C)** sperm DNA fragmentation index (DFI), and **(D)** definite DNA fragmentation index (DDFI). Individual symbols represent individual biological observations (*n* = 3 per group). Data are presented as individual observations with mean ± SEM. Statistical comparisons were performed using one-way ANOVA followed by Tukey's multiple-comparisons test. Statistical significance was considered at *p* < 0.05. ***p < 0.0001; **p < 0.001; *p < 0.01; *p* < 0.05; ns, not significant.

The EMR-exposed group showed a reduction in sperm count compared with the control group (*p* < 0.0001). The EMR + SDR group showed sperm counts that were not significantly different from those of the control group. In contrast, the EMR + AT group also exhibited a lower sperm count than the control group (*p* < 0.0001). The data are presented in Figure 1A.

Exposure to electromagnetic radiation in the EMR group led to a decrease in live spermatozoa and a significant increase in dead spermatozoa relative to the control group (*p* < 0.0001). No significant differences in live and dead spermatozoa were observed between the EMR + SDR and control groups. In the EMR + AT group, the number of live spermatozoa was reduced relative to the control group (*p* < 0.0001), whereas no change was observed in the number of dead spermatozoa. The results are presented in Figure 1B.

### 3.2 Sperm chromatin structure assay (SCSA)

The DFI and DDFI values were higher in the EMR group than in the control group (*p* < 0.0001). The EMR + SDR group did not differ significantly from the control group, whereas the EMR + AT group displayed elevated values (*p* < 0.0001). The EMR + SDR group exhibited a reduction in DNA fragmentation in comparison with the EMR group (*p* < 0.0001). The EMR + AT group did not exhibit significant improvement (Figures 1C,D).

### 3.3 ROS assay

**Figure 2:**
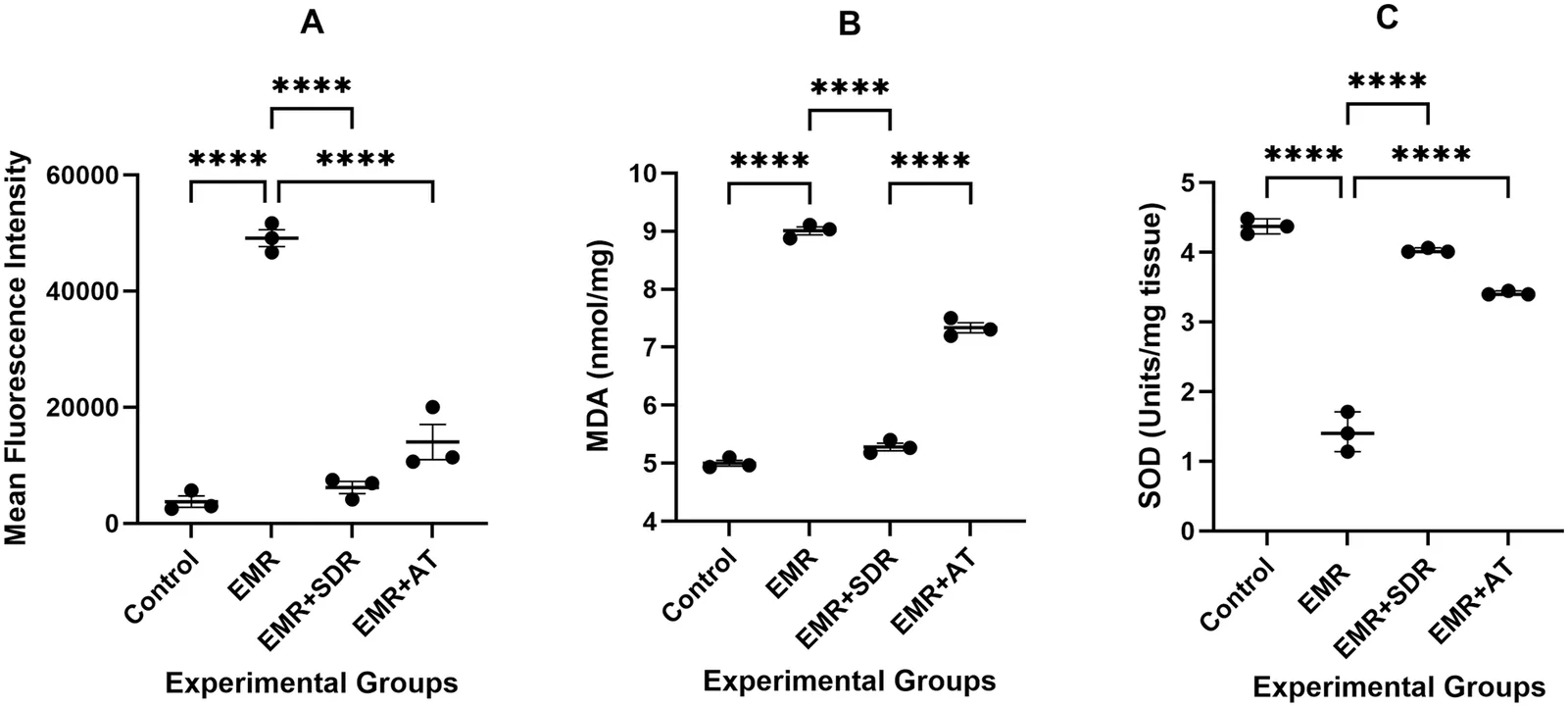
Effects of RF-EMR exposure and swadamshtradi rasayana treatment on oxidative stress and antioxidant defense parameters in Swiss albino mice. **(A)** Reactive oxygen species (ROS), **(B)** malondialdehyde (MDA), and **(C)** superoxide dismutase (SOD). Individual symbols represent individual biological observations (*n* = 3 per group). Data for ROS and MDA are presented as individual observations with mean ± SEM and were analyzed using one-way ANOVA followed by Tukey's multiple-comparisons test. SOD data are presented as individual observations with median and interquartile range (IQR; 25th–75th percentile) and were analyzed using the Kruskal–Wallis test followed by Dunn's multiple-comparisons test. Statistical significance was considered at *p* < 0.05. ***p < 0.0001; **p < 0.001; *p < 0.01; *p* < 0.05; ns, not significant.

ROS levels were significantly higher in the EMR group than in the control group (*p* < 0.0001). The ROS levels in the EMR + SDR group were comparable to those in the control group. ROS levels in the EMR + SDR and EMR + AT groups were reduced compared with the EMR group (*p* < 0.0001). The corresponding data are presented in Figure 2A.

### 3.4 Biochemical assays

#### 3.4.1 SOD activity

Superoxide dismutase activity was significantly lower in the EMR-exposed group than in the control group (*p* < 0.0001). SOD activity in the EMR + SDR group was comparable to that observed in the control group. In the EMR + AT group, SOD activity was lower than that of the control group (*p* < 0.001), although it remained higher than that observed in the EMR group (*p* < 0.0001). SOD activity was lower in the EMR + AT group than in the EMR + SDR group. The results are presented in Figure 2C.

### 3.4.2 Lipid peroxidation (MDA)

Lipid peroxidation was significantly higher in the EMR group than in the control group (*p* < 0.0001). The EMR + SDR group did not differ significantly from the control group with respect to MDA levels, whereas the EMR + AT group showed significantly higher MDA levels than the control group (*p* < 0.0001). A reduction in lipid peroxidation was observed in the EMR + SDR group relative to the EMR group (*p* < 0.0001), with levels lower than those in the EMR + AT group. The data are presented in Figure 2B

## 4 Discussion

The present study aimed to evaluate the radioprotective potential of Swadamshtradi rasayana against RF-EMR-induced spermatozoal anomalies in Swiss albino mice. Experimental and epidemiological studies have demonstrated that exposure to environmental toxicants is associated with impaired male fertility and reduced semen quality (5). The findings indicate that RF-EMR exposure impaired sperm parameters, which may be linked to adverse effects on spermatogenesis. Swadamshtradi rasayana restored sperm count close to control levels, whereas *α*-tocopherol was less effective. These observations align with previous reports suggesting that prolonged exposure to mobile phone radiation may adversely affect semen parameters such as sperm count (41).

Oxidative stress is widely recognized as an important contributor to male infertility. An increase in the concentration of superoxide anion (O₂⁻) leads to increased free radical generation, resulting in oxidative stress and testicular damage (42). RF-EMR exposure reduced sperm viability and compromised membrane integrity. Swadamshtradi rasayana enhanced sperm viability, consistent with protection against oxidative damage, whereas *α*-tocopherol offered only partial protection. Excessive ROS generation contributes substantially to oxidative damage in spermatozoa. Elevated ROS levels can impair sperm function by reducing sperm count, motility, and viability, ultimately leading to male infertility (43). The reduction in ROS levels observed following treatment with Swadamshtradi rasayana may be related to its reported antioxidant properties, whereas the effect of *α*-tocopherol was less pronounced. Mitochondrial dysfunction resulting from excessive ROS production further aggravates sperm damage (10). Spermatozoa are particularly susceptible to oxidative damage because of their high content of polyunsaturated fatty acids, predisposing them to lipid peroxidation (44). Taken together, these observations support oxidative stress as a central mechanism in RF-EMR–induced reproductive damage; however, the precise molecular pathways remain to be clarified.

Although the present study focused on oxidative stress and sperm DNA damage, recent evidence suggests that RF-EMR-induced testicular injury may also involve inflammatory and histopathological alterations. Exposure to 900 MHz RF-EMR has been reported to cause seminiferous tubule damage, interstitial edema, and increased expression of IL-4 and IFN-*γ*, supporting the possibility that alterations in the testicular immune microenvironment may contribute to RF-EMR-induced testicular injury (11). While these parameters were not evaluated in the present study, previous reports suggest that RF-EMR-induced reproductive damage is multifactorial and extends beyond oxidative stress alone. RF-EMR is non-ionizing and lacks sufficient energy to directly induce DNA strand breaks. Instead, RF-EMR-associated DNA damage is considered an indirect consequence of oxidative stress (45). Oxidative stress can adversely affect sperm DNA integrity by inducing both single- and double-stranded DNA breaks (46). In the present study, RF-EMR exposure increased sperm DNA fragmentation. These findings support the interpretation that the observed DNA fragmentation is an indirect consequence of RF-EMR-induced oxidative stress rather than a direct effect of RF-EMR on DNA. Treatment with Swadamshtradi rasayana reduced DNA fragmentation, which may reflect preservation of genomic integrity, whereas *α*-tocopherol was comparatively less effective. Previous studies have likewise reported DNA strand breaks in testicular tissues following RF-EMR exposure (47). In addition, exposure to EMR at frequencies such as 2.45 GHz has been associated with increased DNA damage in reproductive tissues (48). Similarly, RF-EMF exposure at 900 MHz and 1.7 GHz has been reported to induce DNA breakage in cauda epididymal spermatozoa and embryonic stem cells in mice (49). The reduction in DNA damage observed in the present study may reflect decreased oxidative stress, possibly mediated by the antioxidant action of Swadamshtradi rasayana. This protective effect may be linked to the ability of antioxidant compounds to neutralize reactive oxygen species, which may have protected against oxidative DNA strand breaks, thereby preserving chromatin stability and genomic integrity (10).

Several antioxidant interventions have previously been investigated for protection against RF-EMR-induced reproductive toxicity (50). Natural antioxidants such as melatonin, selenium, and vitamin E (*α*-tocopherol) have been reported to ameliorate RF-EMR-induced oxidative stress by reducing reactive oxygen species generation, limiting lipid peroxidation, restoring endogenous antioxidant enzyme activity, and improving sperm quality (51, 52). The present findings are consistent with these reports, supporting the role of antioxidant-based interventions in mitigating RF-EMR-induced reproductive damage. Unlike the single-compound antioxidants evaluated in many previous studies, Swadamshtradi rasayana is a polyherbal formulation, and its observed protective effects are likely to reflect the combined and potentially synergistic actions of its constituent herbs rather than the effect of a single herbal component.

Antioxidant enzymes play an important role in protecting spermatozoa against oxidative stress (53). SOD is one of the key enzymatic antioxidants responsible for neutralizing superoxide radicals and maintaining cellular redox balance (54). RF-EMR exposure was associated with reduced SOD activity, consistent with depletion of endogenous antioxidant defenses. Treatment with Swadamshtradi rasayana increased SOD activity, accompanied by improved antioxidant status, and may have contributed to enhancement of the antioxidant defense system, whereas *α*-tocopherol showed only partial improvement. Previous studies have reported that increased production of reactive superoxide ions induces oxidative stress, which may lead to inactivation of antioxidant enzymes, including SOD (54).

Lipid peroxidation is another important marker of oxidative stress in spermatozoa. Increased MDA levels, a by-product of lipid peroxidation, have been linked to membrane damage and impaired sperm function (55). The results showed that RF-EMR exposure increased lipid peroxidation, which may reflect oxidative membrane damage. Administration of Swadamshtradi rasayana reduced lipid peroxidation levels, which may have contributed to the protection of sperm membrane integrity, whereas *α*-tocopherol showed a comparatively smaller reduction. Increased ROS production can promote lipid peroxidation, leading to sperm membrane damage and impaired sperm function (56).

The observed effects of Swadamshtradi rasayana may be attributed to the synergistic antioxidant properties of its constituent herbs, namely *Phyllanthus emblica, Tinospora cordifolia, and Tribulus terrestris.* The observed reduction in ROS levels and lipid peroxidation, together with restoration of SOD activity, is consistent with the antioxidant effects of Swadamshtradi rasayana. *Phyllanthus emblica* contains bioactive compounds such as emblicanin A and B, which possess potent free radical scavenging properties. Previous studies have shown that its extracts improve sperm parameters and protect against oxidative and structural damage, including increased MDA levels, seminiferous tubule damage, and reduced testosterone levels (57, 58). *Tinospora cordifolia* possesses potent free radical scavenging properties and has been reported to reduce ROS and lipid peroxidation while enhancing antioxidant defense systems (59, 60). *Tribulus terrestris* is widely recognized for its aphrodisiac and androgen-enhancing properties, primarily due to the presence of steroidal saponins such as protodioscin (61). Experimental studies have demonstrated that it improves sperm parameters, including count, motility, and viability, through modulation of intracellular signaling pathways (24, 62). Collectively, these findings suggest that the observed protective effects may be partly attributable to the combined antioxidant properties of these herbs.

## 5 Conclusion

Exposure to RF-EMR resulted in reduced sperm parameters and increased levels of ROS, lipid peroxidation, and DNA damage. Administration of Swadamshtradi rasayana was associated with radioprotective effects against RF-EMR–induced sperm damage, as evidenced by improved sperm count, viability, and antioxidant status. Biochemical and molecular analyses further indicated that Swadamshtradi rasayana showed greater improvement than *α*-tocopherol in attenuating oxidative stress. Overall, these findings suggest that Swadamshtradi rasayana may help preserve sperm DNA integrity and attenuate oxidative stress–mediated damage following RF-EMR exposure.

### 5.1 Limitations and future prospects

Despite incorporating advanced molecular and biochemical assessments such as reactive oxygen species (ROS) estimation, antioxidant enzyme activity, and sperm DNA fragmentation analysis, the present study has certain limitations. Functional evaluation of sperm was limited to sperm count and viability, while other parameters such as motility, morphology, and kinematic analysis were not assessed, which could have provided additional insight into sperm functional competence.

Furthermore, histopathological evaluation of the testis was not performed. Assessment of seminiferous tubule architecture, germinal epithelium integrity, and Leydig cell morphology would have provided complementary structural evidence to support the observed biochemical findings and further substantiate the protective effects of the formulation on testicular tissue. Incorporation of hormonal profiling, including serum testosterone, luteinizing hormone (LH), and follicle-stimulating hormone (FSH), could have provided additional insight into whether the observed improvements were accompanied by preservation of reproductive endocrine function. Although exposure conditions were standardized across groups, the absence of comprehensive dosimetric characterization, including specific absorption rate (SAR), power density, field strength, and exposure uniformity, may limit precise quantification of RF-EMR exposure and restrict comparison with previous RF-EMR studies and the reproducibility of the exposure conditions. In addition, the limited sample size and use of a single dose level without dose–response assessment or detailed pharmacological validation may limit the generalizability and interpretation of the findings. Although the herbal ingredients were botanically authenticated prior to formulation, detailed phytochemical characterization of the formulation, including HPLC or LC-MS chromatographic profiling, was not performed. Furthermore, the systemic bioavailability of the bioactive constituents was not evaluated in the present study.

Future studies incorporating phytochemical characterization and pharmacokinetic analyses are warranted to better elucidate the pharmacological mechanisms underlying the observed radioprotective effects. Future clinical investigations are needed to confirm the applicability and translational relevance of these findings in human populations. Future studies incorporating comprehensive sperm functional assessment, hormonal profiling, histopathological evaluation of testicular tissue, detailed RF-EMR dosimetric characterization, larger sample sizes, and multiple dose levels are warranted to validate and extend these findings.

## Data Availability

The raw data supporting the conclusions of this article will be made available by the authors, without undue reservation.
